# Biomechanical behavior of implants with different diameters in relation to simulated bone loss— an in vitro study

**DOI:** 10.1007/s00784-023-05199-5

**Published:** 2023-08-23

**Authors:** Tobias Graf, Jan-Frederik Güth, Josef Schweiger, Kurt-Jürgen Erdelt, Daniel Edelhoff, Michael Stimmelmayr

**Affiliations:** 1 Department of Prosthetic Dentistry, Center for Dentistry and Oral Medicine, Goethe University Frankfurt, Theodor-Stern-Kai 7, 60596 Frankfurt am Main, Germany; 2 Department of Prosthetic Dentistry, University Hospital, Ludwig-Maximilians-University Munich, Goethestraße 70, 80336 Munich, Germany

**Keywords:** Bone level, Dental implants, Fatigue testing, Implant-abutment interface, Implant diameter, Stability

## Abstract

**Objectives:**

Bone resorption around implants could influence the resistance of the implant abutment complex (IAC). The present in vitro study aimed to assess the stability to static fatigue of implants presenting different levels of bone losses and diameters.

**Materials and methods:**

Ninety implants with an internal conical connection with 3 different implant diameters (3.3 mm (I33), 3.8 mm (I38), and 4.3 mm (I43)) and 3 simulated bone loss settings (1.5 mm (I**_**15), 3.0 mm (I**_**30), and 4.5 mm (I_45) (*n* = 10)) were embedded and standard abutments were mounted. All specimens were artificially aged (1,200,000 cycles, 50 N, simultaneous thermocycling) and underwent subsequently load-to-fracture test. For statistical analysis, Kolmogorov–Smirnov test, Kruskal–Wallis test, and Mann–Whitney *U* test (*p* < 0.05) were applied.

**Results:**

All test specimens withstood the artificial aging without damage. The mean failure values were 382.1 (± 59.2) *N* (I3315), 347.0 (± 35.7) *N* (I3330), 315.9 *N* (± 30.9) (I3345), 531.4 (± 36.2) *N* (I3815), 514.5 (± 40.8) *N* (I3830), 477.9 (± 26.3) *N* (I3845), 710.1 (± 38.2) *N* (I4315), 697.9 (± 65.2) *N* (I4330), and 662.2 *N* (± 45.9) (I4345). The stability of the IACs decreased in all groups when bone loss inclined. Merely, the failure load values did not significantly differ among subgroups of I43.

**Conclusions:**

Larger implant diameters and minor circular bone loss around the implant lead to a higher stability of the IAC. The smaller the implant diameter was, the more the stability was affected by the circumferential bone level.

**Clinical relevance:**

Preserving crestal bone level is important to ensure biomechanical sustainability at implant systems with a conical interface. It seems sensible to take the effect of eventual bone loss around implants into account during implant planning processes and restorative considerations.

## Introduction

Implant-supported restorations are a common treatment option, and their application increased over the last years; for example, from 2005 up to 2014, the number of implant-supported dental prostheses in Germany tripled. So, the desire of patients seems to loom large [1]. Implants and the resulting additional implant prosthetic restoration options significantly boost the patients’ quality of life and their satisfaction [2, 3]. However, sufficient and inflammation-free hard- and soft-tissue conditions are a “conditio sine qua non” for long-term stability and success.

Meanwhile, there are numerous implants available on the dental market that are smaller than the “standard diameter” of 3.75 mm. In a literature review by Al-Johany et al., diameters between 1.8 and 7.0 mm were reported. Nevertheless, numerous terms and definitions can be found in the literature in relation to “narrow”, “standard”, and “wide” diameters. At the same time, the distinction between these classifications is not defined and differs depending on authors and manufacturers [4]. The same problem of definition exists in the classification of implant length. The terms “short”, “regular”, and “long” are frequently used in the literature, despite the fact that the boundaries overlap [4]. Compared to previous years, the trend is to place shorter and thinner implants. Avoiding extensive bone augmentation and expanding the range of indications to insert implants might be the most common reasons for this trend [5].

“Short” implants seem to be an effective treatment alternative for the atrophic posterior ridge, as failure rates did not differ compared to longer implants combined with bone augmentation [6, 7]. According to Altbait et al., this therapy may even have less marginal bone loss and fewer postoperative complications [6]. For implant diameters between 3.0 and 3.5 mm, there are also no statistically significant lower survival rates in the first years compared to those with larger diameters. For even smaller diameters, the survival rate decreased significantly. Yet, valid long-term data expanding 5 years are not available [8].

In some cases, fractures have been clinically reported, especially of implants with reduced diameter (3.0–3.7 mm) [9, 10]. This biological complication inevitably leads to a removal of the implant and its superstructure. The finding is confirmed in a review by of Gealh et al. who reported that the implant diameter has a direct effect on a potential implant fracture [11]. Particularly thin implants in the molar region could be prone to this, as maximum masticatory forces of up to 740 N have been reported and can be 4 times higher compared to the anterior region [12, 13]. Furthermore, tactility is limited due to the absence of Sharpey’s fibers, which results in permanently increased chewing forces and affects the materials of the implants themselves as well as their superstructures [14].

This investigation aims to examine how different implant diameters influence the resistance of the implant shoulders and the conical implant-abutment complex (IAC) in general. Allum et al. has already found in a similar in vitro study that implant diameters demonstrated a major impact on their ability to withstand load [15]. Since periimplantitis accompanied by crestal bone loss around implants is nowadays one frequent biological complication, the circumferential bone-loss level around the implant is considered as a further parameter in the present investigation. Both variables will be combined in the present study. Varied leverage effects at the IAC might have an effect on failure values of the components. Further conclusions about potential failure modes should be observed. The first hypothesis is that the stability of the IAC is not influenced by the implant diameter. Secondly, we postulate that the circumferential bone level does not affect the resistance of the IAC.

## Material and methods

### Specimen fabrication

**Fig. 1:**
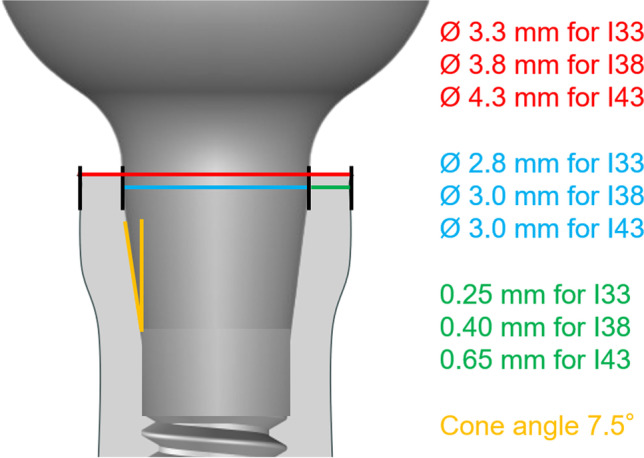
Illustration of the implant system (not to scale) in the present study used with their dimensions for I33, I38, and I43

**Fig. 2:**
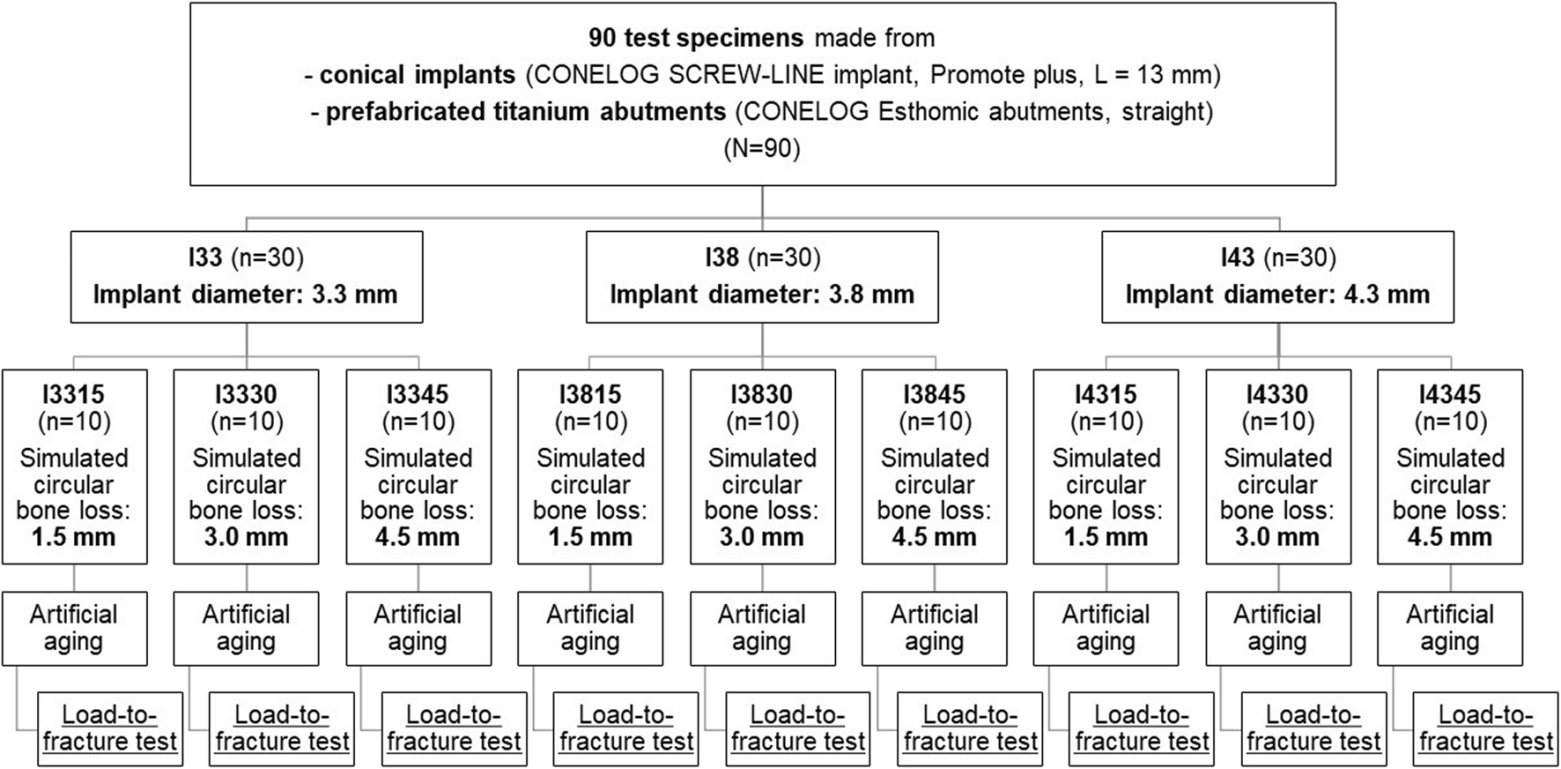
Experimental sequence

**Fig. 3:**
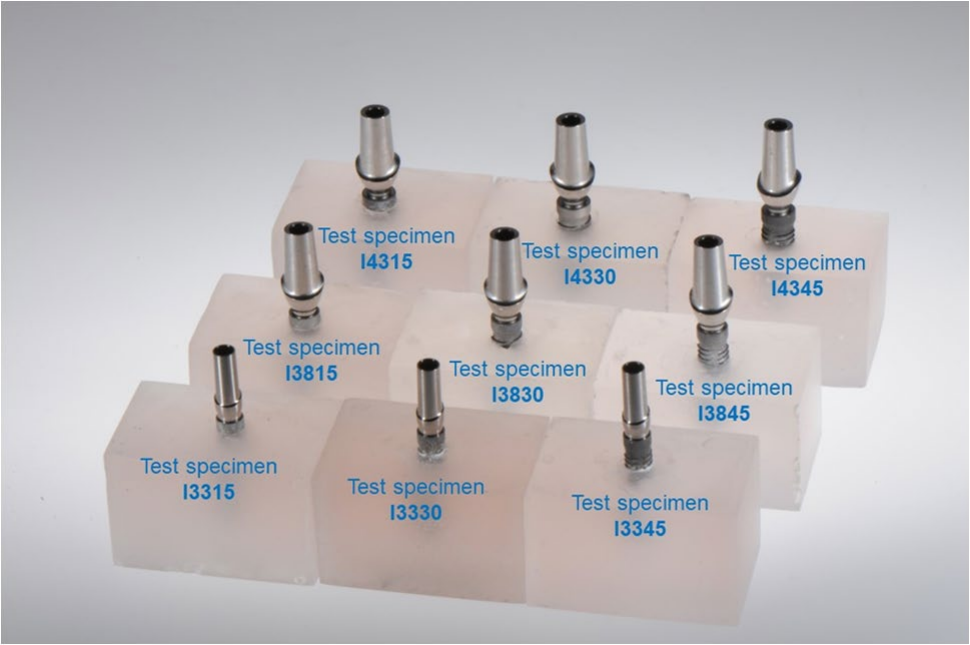
Test specimens with their diameters (3.3, 3.8, and 4.3 mm) and simulated bone loss (1.5, 3.0, and 4.5 mm) representing all 9 groups

The test specimens were produced according to ISO standard 14801:2017. Therefore, 90 implants with conical internal connection were embedded in self-curing laminating resin blocks (DPC laminating resin LT 2, Duroplast-Chemie Vertriebs GmbH, Neustadt/Wied, Germany) with an E-modulus over 3.0 GPa following ISO standard (*N* = 90). The implants were Conelog implants (CONELOG SCREW-LINE implant, Promote plus, L = 13 mm, Camlog Biotechnologies AG, Basel, Switzerland) with diameters “d” of 3.3 mm (I33), 3.8 mm (I38), and 4.3 mm (I43) (Fig. 1). For each diameter, 3 different levels of circular bone loss were simulated. 10 implants per test group (*n* = 10) were embedded 1.5 mm, 3.0 mm (ISO standard), and 4.5 mm below their nominal bone levels that should simulate the respective bone loss (Fig. 2). For each diameter, 3 different situations with various circular bone losses “h” were created, namely 1.5 mm (I_15), 3.0 mm (I_30), and 4.5 mm (I_45) resorption. Prefabricated titanium abutments (abutment height: 9.0 mm; CONELOG Esthomic abutments, straight, Camlog Biotechnologies AG, Basel) were screwed onto the implants with a controlled torque of 20 Ncm considering the manufacturer’s instructions (Figs. 2 and 3). Sample size of the subgroups was chosen in concordance with preceding studies presenting a similar experimental setup.

### Artificial aging and load-to-fracture test

All test specimens underwent artificial aging in a chewing simulator (CS-4 chewing simulator; SD Mechatronik, Feldkirchen-Westerham, Germany) with 1,200,000 cycles and an applicated force of 50 N. The axis of the implants to the stainless steel stylus of the chewing simulator (diameter 5.0 mm) was 30°. Simultaneously, thermocycling with 10,000 alternating cold and hot water cycles (10,000 cycles between 5 °C and 55 °C; dwelling time 60 s; distilled water) was conducted. After each 100,000 cycles, the specimens were visually inspected for damage or failure (e.g. screw loosening, screw fracture, abutment fracture, and implant fracture).

**Fig. 4:**
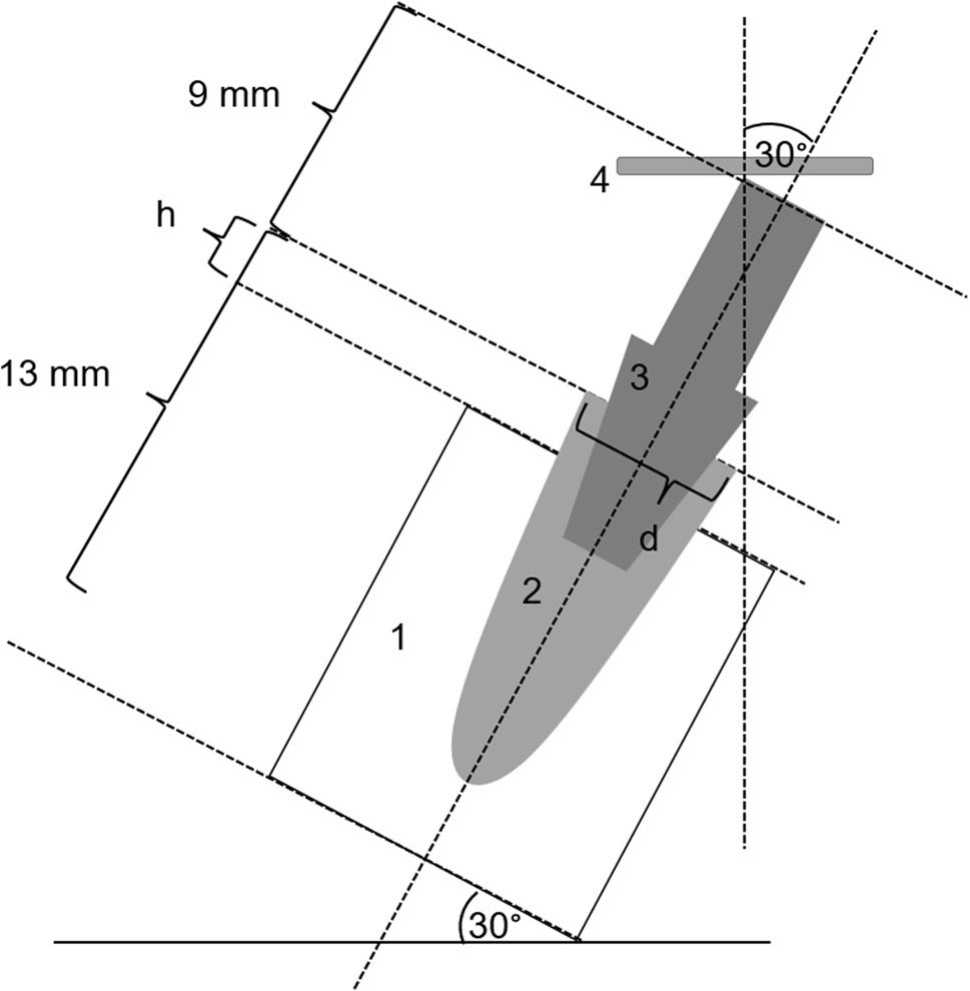
Illustration of load-to-fracture test (1, resin block; 2, embedded implant with group-specific implant diameter “d” and simulated bone loss “h”; 3, abutment; 4, steel plate)

Following, all intact specimens were loaded until failure in a universal testing machine (Zwick UPM 1445; Zwick GmbH & Co. KG, Ulm, Germany). The force application was performed by a steel plate at 30° off-axis according to ISO standard with loading speed of 0.5 mm/min (Fig. 4). The failure load values were defined by a sudden decrease of more than 20% in the force-path diagram or a 2.0 mm deflection of the steel plate. However, all test specimens were further loaded up to 2.0 mm deflection of the steel plate even if their maxima were reached prior. The failure modes around the IAC were examined and documented after unscrewing the implant-abutment components.

### Statistics

IBM SPSS Statistics 25 for Windows (International Business Machines Corporation, Armonk, NY USA) was used for statistical analysis of the data. By means of descriptive statistics, the failure load means and standard deviations of all material groups were calculated. The mean fracture load values of the test groups were checked for normal distribution using Kolmogorov–Smirnov test. Based on this, significant differences were tested with one-way analysis of variance (ANOVA Scheffè-method (post-hoc test)) or Kruskal–Wallis test and Mann–Whitney *U* test. The significance level was set to *p* < 0.05.

## Results

**Fig. 5:**
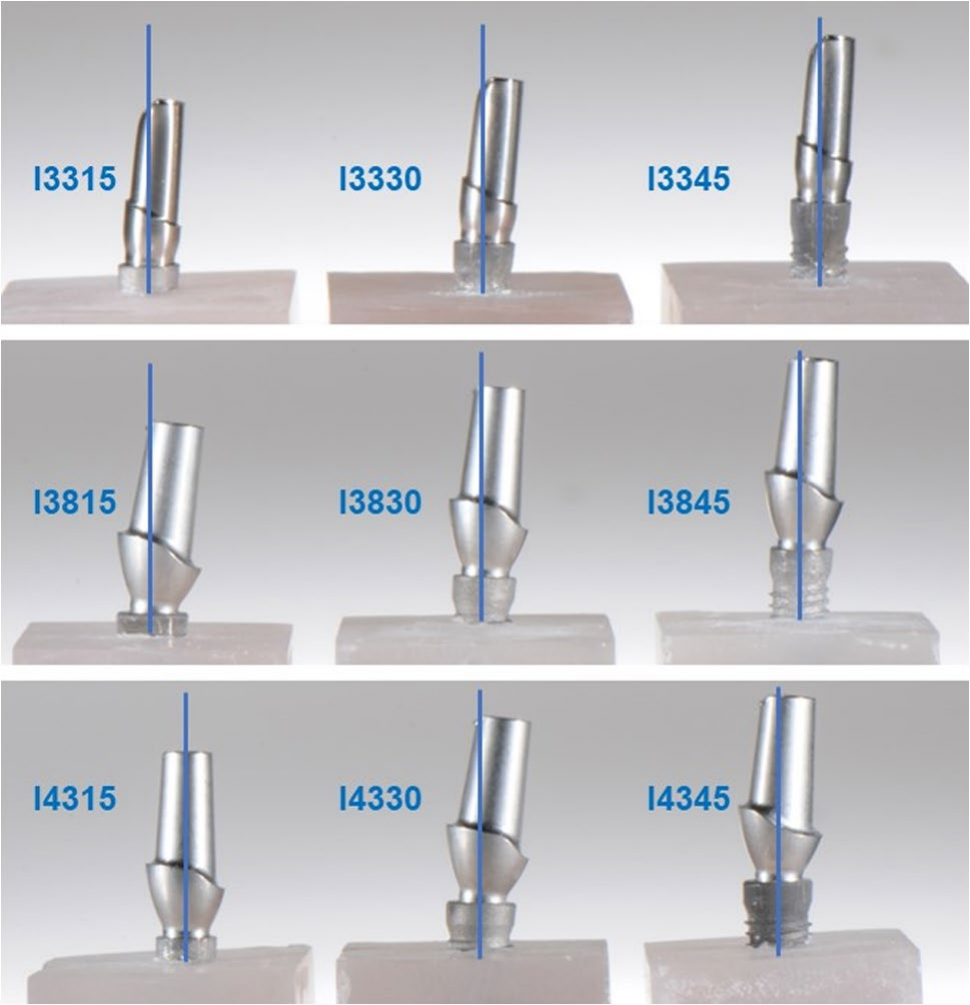
Exemplary deflections of all test groups after load-to-fracture test

All 90 test specimens survived the artificial aging without any complications. The mean failure load values of I33, I38, and I43 were 348.3 ± 50.3 N, 507.9 ± 40.7 N, and 690.1 ± 53.4 N, respectively (Table 1). A normal distribution of these values was found so that significant differences between the test groups were evaluated using the ANOVA Scheffè-method (post-hoc test). I33, I38, and I43 showed statistical significant differences (*p* ≤ 0.001). The failure mode during the fracture load test was analogous for all test specimens and characterized by a visual deflection of the abutments and deformation of the implant shoulders (Fig. 5). No other failure modes could be detected.

**Table 1:** Group-specific mean failure values including maxima and minima

	Mean failure values [N]	Standard deviation [N]	Maximum [N]	Minimum [N]
I33	348.3	50.3		
I3315	382.1	59.2	495.0	313.0
I3330	347.0	35.7	428.0	316.0
I3345	315.9	30.9	372.0	283.0
I38	507.9	40.7		
I3815	531.4	36.2	605.0	494.0
I3830	514.5	40.8	581.0	451.0
I3845	477.9	26.3	518.0	440.0
I43	690.1	53.4		
I4315	710.1	38.2	768.0	636.0
I4330	697.9	65.2	810.0	607.0
I4345	662.2	45.9	721.0	572.0

**Fig. 6:**
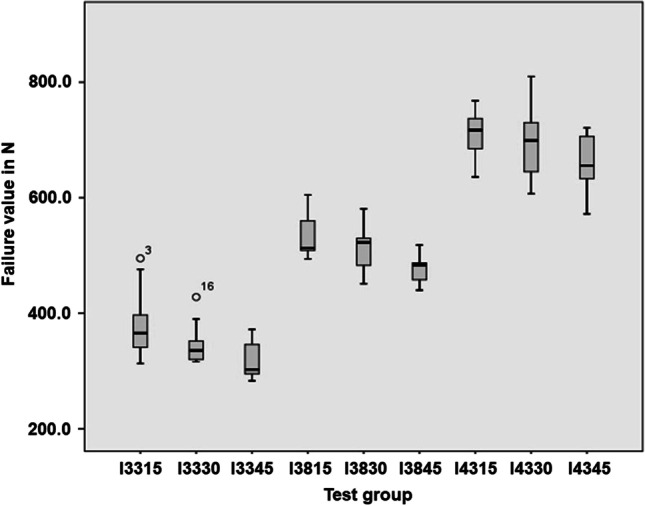
Boxplot diagram of all subgroups after load-to-fracture test

The mean failure load values of the subgroups including deviations are 382.1 ± 59.2 N (I3315), 347.0 ± 35.7 N (I3330), 315.9 ± 30.9 N (I3345), 531.4 ± 36.2 N (I3815), 514.5 ± 40.8 N (I3830), 477.9 ± 26.3 N (I3845), 710.1 ± 38.2 N (I4315), 697.9 ± 65.2 N (I4330), und 662.2 ± 45.9 N (I4345) (Table 1) and are visualized by boxplot diagrams in Fig. 6. Since the subgroups I3345 and I3815 (*p* = 0.011 and *p* = 0.014) did not show a normal distribution, significant differences between the subgroups were determined using the Kruskal–Wallis test (Table 2) and Mann–Whitney *U* test (Table 3). There were significant differences between subgroups I3315 and I3345, I3330 and I3345, I3815 and I3845, I3830 and I3845, I4315 and I4330, I4315 and 4345, as well as I4330 and I4345 (Tables 2, 3).

**Table 2:** *P*-values of Kruskal Wallis test for subgroups of I33, I38, and I43

	**I33**		
	I3315	I3330	I3345
*P*-value	*p* = 0.007*		
	**I38**		
	I3815	I3830	I3845
*P*-value	*p* = 0.008*		
	**I43**		
	I4315	I4330	I4345
*P*-value	*p* = 0.107		

*Subgroups statistically showed significant differences

**Table 3:** *P*-values of Mann–Whitney *U*-test showing a pairwise comparison of the respective subgroups I33 and I38

	I33					
	I3315	I3330	I3315	I3345	I3330	I3345
*P*-value	*p* = 0.105		*p* = 0.003*		*p* = 0.043*	
	**I38**					
	I3815	I3830	I3815	I3845	I3830	I3845
*P*-value	*p* = 0.529		*p* = 0.002*		*p* = 0.029*	

*Subgroups statistically showed significant differences

## Discussion

The null hypotheses cannot be accepted: the failure load values clearly depend on the diameter of the implants. Simultaneously, the circumferential bone level around the implants was a crucial factor since the stability values significantly differed depending on the simulated bone loss, particularly in implants with smaller diameter. The effect of enhancements of the resistance-to-fracture with increasing implant diameter is also described in other studies [15–18] and is absolutely plausible from a biomechanical point of view. So, Allum et al. urge caution when using implants with a diameter of 3.0 mm or less [15]. Additionally, the design of the interface between the implant and the abutment plays a key role for the (long-term) success without failures of the IAC [17–20]; Lee et al. even attributed in a FE analysis higher relevance to this factor than to the implant diameter [17].

Other in vitro studies also demonstrated that stability values decrease with progressive bone loss [21, 22]. Furthermore, Manzoor et al. recognized that failure modes changed with increased bone resorption. Up to 1.5 mm loss, increased screw, and abutment fractures were observed in the load-to-fracture test, whereas the simulated bone loss of 3.0 and 4.5 mm lead to fractures of the implant body [22]. This shift of complication patterns could not be confirmed in the present study, although the specimens were loaded over their maxima up to a deflection of 2.0 mm—a not clinically relevant level. In this context, Gehrke et al. mentioned that the design of IAC might be pivotal for their performance and resistance when the bone level changed [21].

After extensive research, a combination of these two parameters—implant diameter and bone loss—within one study is not yet available in the literature despite the high relevance of both factors in combination and considering the problematic comparability between different studies. Looking more closely at the present results when comparing the subgroups, it could be shown that the failure of implant platforms did not differ significantly with bone resorption of up to 3.0 mm, independent of the implant diameter tested. Secondly, bone loss of up to 4.5 mm at implants with diameters of 4.3 mm did not significantly affect their resistance to fracture. Consequently, it can be implied that thicker implant walls can better withstand high extra-axial forces even when the bone level is heavily reduced over time.

A larger implant diameter might also lead to a certain safety factor in terms of implant shoulder resistance if peri-implantitis therapy is performed by implantoplasty. In vitro studies of Camps-Font et al. and Chan et al. showed that implantoplasty in implants with a diameter of 3.75 mm or smaller produces a decrease in fracture resistance [23, 24]. Even if the effects depend on the respective implant system and the IAC, clinicians should be aware that the implants after implantoplasty therapy might be more prone to fail, especially if hexagonal and conical connections are used [23, 25].

In the present study, implants with an internal conical connection were used. The connection design seems to be crucial for the force distribution on the IAC. Varying force distribution could lead to peri-implant bone loss such as peri-implant inflammation [26]. A comparison of their in vitro stability to butt-joint–connected implant systems shows that the latter can withstand a significantly higher load [18, 19]. However, the interface from the implant shoulder and the abutment is closer to the bone when no platform switch exists. Platform switching seems to be successful in reducing bone loss around dental implants [27, 28].

Periodontal diseases are significantly increasing, especially in patients over 50 years of age [29, 30]. Peri-implantitis manifests in similar ways as periodontitis in dentate areas. The reduced attachment level results in unfavorable leverage ratios between the crown and the implant, especially in the area of the sensitive IAC. Morgan et al. showed that implant body fractures can even occur in vivo, especially when there is a circular bone resorption around the implant and thus, osseous support is missing [31].

Furthermore, to achieve long-term survival of implants, inflammation-free conditions around the implants must be ensured. However, biological risk factors trigger peri-implantitis and thus bone resorption is not entirely preventable, as many factors seem to be causative. These include patients with a previous history of chronic periodontitis, poor plaque control, and no regular aftercare. However, nicotine abuse, diabetes, or genetic predispositions are also potential risk factors. Other factors, such as the presence of submucosal cement after restoration placement, the absence of peri-implant keratinized mucosa, micromovements along the IAC, or a position of the implants that makes it difficult to manage oral hygiene and care, are associated with direct peri-implantitis [32–34].

For clinicians, attention should be paid to regular patient recall appointments as well as individualized risk-based “supportive periodontal therapy” (SPT) in the presence of periodontitis [35, 36]. Given the fact that progressive crestal bone-loss around implants without clinical signs of soft tissue inflammation do not occur mostly [32], the risk of bone loss can be minimized.

This study showed that thicker implants seem to have a protective effect in the presence of bone loss. Therefore, considerations should already be given to this during implant planning phase as one factor, in case bone resorption may occur many years later.

The study design is similar to other in vitro studies investigating the stability and longevity of implants and their superstructures. Artificial aging should correspond to an in-vivo simulation of about five years [37]. This procedure seems not to reduce stability of the implant shoulder significantly. However, this process seems to have an influence primarily on the screw connection between implant and abutment [18]. From the present study, it can be concluded that the conical internal connection generates a reliable connection between the implant and the abutment during the five-year in vitro aging, regardless of the implant diameter, since no screw loosening was observed.

The ISO 14801 standard sets general parameters for fatigue testing of enossal dental implants, such as loading angle to the test specimens, embedding material, or simulated bone loss. Opposed to the ISO standard, no semi-sphere-shaped “loading abutments” were applied in the test setup. From the biomechanical point of view, the shape of the abutments should not significantly bias the findings and conclusions of the study especially since the load was applied at a 30° angle. The continuous force increase during the fracture load test does not represent the intraoral clinical conditions, but instead is reproducible. Nevertheless, in vitro studies are difficult and not useful to compare with other studies concerning their absolute failure values, respectively stability values. There are—among other things—two reasons for this: Firstly, the failure criteria for the fracture load test are not standardized, and secondly, the implant systems are usually different with implant-abutment geometries and different diameters [18–25]. Nevertheless, the presented study shows significant findings that are relevant in regards to the selection of implants and restorations, especially in terms of their behavior under unideal conditions over time.

## Conclusion

Within the limitations of the study, it can be concluded that maintaining crestal bone levels is important to ensure biomechanical sustainability at conical IACs. Against the background of achieving a long, complication-free implant survival, it seems sensible to take—among other parameters—these aspects into account in the previous implant planning:

1. Larger implant diameters and minor bone loss around the implant shoulder showed higher stability values at IAC.
2. The larger the implant diameter, the less resistance at IAC seems to be affected by bone loss.

Further studies evaluating different IACs in this regard are necessary.

## Data Availability

The data that support the findings of this study are available from the corresponding author [T.G.] upon reasonable request.
